# Effects of vitrification on nuclear maturation, ultrastructural changes and gene expression of canine oocytes

**DOI:** 10.1186/1477-7827-8-70

**Published:** 2010-06-22

**Authors:** Bongkoch Turathum, Kulnasan Saikhun, Parisatcha Sangsuwan, Yindee Kitiyanant

**Affiliations:** 1 Department of Anatomy, Faculty of Science, Mahidol University, Rama 6 Road, Bangkok 10400, Thailand; 2 Institute of Molecular Biosciences, Mahidol University, Nakhon Pathom 73170, Thailand

## Abstract

**Background:**

Cryopreservation of oocytes, which is an interesting procedure to conserve female gametes, is an essential part of reproductive biotechnology. The objective of the present study was to investigate the effects of vitrification on nuclear maturation, ultrastructural changes and gene expression of canine oocytes.

**Methods:**

Immature oocytes (germinal vesicles) isolated from ovaries of normal bitches (> 6 months of age) were either vitrified in open pulled straw (OPS) using 20% ethylene glycol (EG) and 20% dimethyl sulfoxide (DMSO) as vitrification solution or exposed to vitrification solution without subjected to liquid nitrogen. After warming, oocytes were investigated for nuclear maturation following in vitro maturation (IVM), ultrastructural changes using transmission electron microscopy (TEM) and gene expression using RT-PCR. Fresh immature oocytes were used as the control group.

**Results:**

The rate of resumption of meiosis in vitrified-warmed oocytes (53.4%) was significantly (P < 0.05) lower than those of control (93.8%) and exposure (91.4%) groups. However, there were no statistically significant differences among groups in the rates of GV oocytes reaching the maturation stage (metaphase II, MII). The ultrastructural alterations revealed by TEM showed that cortical granules, mitochondria, lipid droplets and smooth endoplasmic reticulum (SER) were affected by vitrification procedures. RT-PCR analysis for gene expression revealed no differences in HSP70, Dnmt1, SOD1 and BAX genes among groups, whereas Bcl2 was strongly expressed in vitrified-warmed group when compared to the control.

**Conclusion:**

Immature canine oocytes were successfully cryopreserved, resumed meiosis and developed to the MII stage. The information obtained in this study is crucial for the development of an effective method to cryopreserve canine oocytes for establishment of genetic banks of endangered canid species.

## Background

A major obstacle for the development of assisted reproductive technologies in canines is the low percentage of oocytes reaching the maturation stage (i.e., metaphase II, MII) following IVM. In contrast to most of other mammals that oocytes are at the MII stage when ovulated, canine oocytes released from ovaries are at the prophase I stage of the first meiotic division and they subsequently completed nuclear maturation within 60-72 h in the oviduct [1]. Several studies have been made to improve the rates of oocyte maturation in vitro, however, little progress has been achieved and usually less than 20% of canine oocytes complete nuclear maturation [2,3]. Although the low efficiency of IVM of bitch oocytes remaining unresolved, the development of oocyte cryopreservation is important for establishing genetic banks as well as for developing applications for conservation of endangered canid species [2]. The first successful IVF producing live offspring from cryopreserved mouse oocytes frozen and stored in liquid nitrogen was reported in 1977 [4]. Subsequently, successful cryopreservation of oocytes has been achieved in other mammalian species [5,6] including human [7,8]. However, there is no report on the cryopreservation of canine oocytes.

Previous studies reported success in cryopreservation of bovine oocytes but mature (MII stage) oocytes were susceptible to cooling damage resulting in disruption of meiotic spindle and chromosome [9]. Ultrastructural studies on vitrified bovine oocytes have revealed that intercellular communication between the cumulus cells and oocyte might have been interrupted and that the zona pellucida might have been modified by premature cortical granule release [10]. Ultrastructural alterations of the cytoskeleton, mitochondria, cortical granules and nucleoli have also been observed in bovine oocytes [11,12]. Structural changes of vitrified oocytes have also been observed in porcine [13] and human oocytes [14]. Immature oocytes in which organization of the meiotic spindle did not develop may be an alternative source for genetic banks. Therefore, the aim of this study was to investigate the effects of vitrification on nuclear maturation, ultrastructural changes and gene expression on vitrified-warmed immature canine oocytes.

## Methods

### Chemicals and media

All chemicals in this study were purchased from Sigma Chemical Company (Sigma, St. Louise, MO, USA), unless indicated otherwise. Media was prepared once a week, filtered (0.2 μm, Sartorius, Minisart, CA, USA) and kept in sterile bottles. Synthetic oviductal fluid (SOF) cultured media was incubated at 38.5°C under 5% CO_2 _in air at least 4 h before use.

### Collection of oocytes

Ovaries were obtained from normal bitches of various breeds at various ages (> 6 months old) by ovariohysterectomy at the veterinary clinic of the Veterinary Public Health Division, Bangkok Metropolitan Administration. Ovaries were placed in 0.9% NaCl (containing 100 IU/ml penicillin) and transported to the laboratory (at 25-32°C) within 2-4 h after removal. Ovaries were washed three times in 0.9% NaCl containing 100 IU/ml penicillin. To collect cumulus-oocyte complexes (COCs), ovaries were sliced repeatedly in Petri dishes containing TCM 199 (Invitrogen, Carlsbad, CA, USA) supplemented with 25 mM HEPES, 0.1% polyvinylalcohol, 0.1 mM glutamine, 2.5 mM sodium pyruvate and 1% penicillin-streptomycin. Cumulus-oocyte complexes were washed and graded under a stereomicroscope (200×) using criteria based on the uniformity of ooplasm and cumulus cell complement, as previously described [15]. Grade 1 oocytes were surrounded with more than five layers of compact cumulus cells and had homogeneous dark cytoplasm. Grade 2 oocytes were surrounded by three to five layers of compact cumulus cells and had homogeneous dark cytoplasm. Grade 3 oocytes were partially surrounded by cumulus cells and lacked homogeneous cytoplasm. Grade 4 oocytes were denuded (without surrounding cumulus cells) and lacked homogeneous cytoplasm. Cumulus-oocyte complexes with more than three layers of cumulus cells with dark pigment oocyte cytoplasm (grade 1 and grade 2) were selected for the experiments.

### Vitrification and warming of immature oocytes

The procedures for cryopreservation of oocytes were performed at room temperature. Immature oocytes (germinal vesicle stage) were exposed to the holding medium (HM) (TCM 199 supplemented with 20 mM Hepes and 20% FBS) for 5 min and then equilibrated in HM+4% EG for 5 min. They were exposed to HM+10% EG+10% DMSO for 1 min and then placed in 2-3 μl drop of the vitrification solution (HM+20% EG+20% DMSO+0.5 mM sucrose) for 30 sec. The COCs were then loaded in OPS straws (five oocytes per straw), plunged directly into the liquid nitrogen and stored for 7 days. Warming was done by immersing the tip of OPS straw into 1 ml of HM+0.3 mM sucrose at 37°C for 1 min. The oocytes were directly expelled into the medium after the vitrified medium became liquid. The oocytes were transferred into HM+0.15 M sucrose for further rehydration. They were then washed and incubated in holding medium for 5 min before further evaluation.

### Morphological assessment of vitrified-warmed oocytes

**Figure 1:**
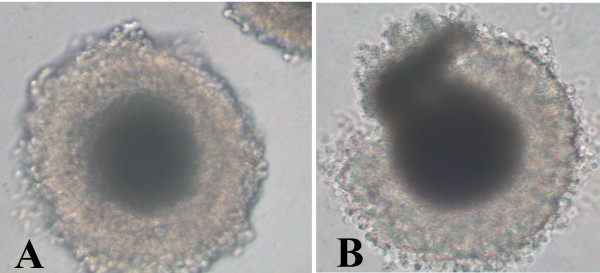
**Morphology of vitrified-warmed oocytes after warming (200×)**. A) Normal oocyte with compacted cumulus cells, symmetrical shape and no signs of lysis. B) Abnormal oocyte with damaged cumulus cells, ruptured zona pellucida and leakage of cytoplasm.

Vitrified-warmed oocytes were assessed for viability according to their morphology. The COCs surrounded with compacted cumulus cells, of symmetrical shape and showing no signs of lysis were classified as of normal morphology (Figure 1A). Contrarily, COCs with damaged cumulus cells, ruptured zona pellucida or leakage of cytoplasm were classified as abnormal oocytes (Figure 1B). Only vitrified-warmed oocytes with normal morphology were subjected to in vitro maturation.

### In vitro maturation

The COCs were cultured in SOF supplemented with 40 ng/ml epidermal growth factor (EGF) [3]. Twenty COCs were cultured for 48 h in a four-well Petri dishes containing 500 μl SOF under mineral oil in each well. The dishes were held in an incubator at 38.5°C in a humidified atmosphere of 5% CO_2 _in air.

### Experimental design

#### Experiment I: Nuclear maturation of vitrified-warmed canine oocytes

Cumulus-oocyte complexes were divided into 3 groups: 1) control, 2) exposure; oocytes were exposed to vitrification media without loading into straws and storage in liquid nitrogen, and 3) vitrified-warmed; oocytes were vitrified, loaded into straws, stored in liquid nitrogen for 7 days and warmed at 38.5°C. Cumulus-oocyte complexes were cultured in SOF for 48 h at 38.5°C. Following 48 h culture, they were denuded, fixed and stained with Hoechst 33342. They were then evaluated for the meiotic stage under a fluorescence microscope.

#### Assessment of nuclear maturation

**Figure 2:**
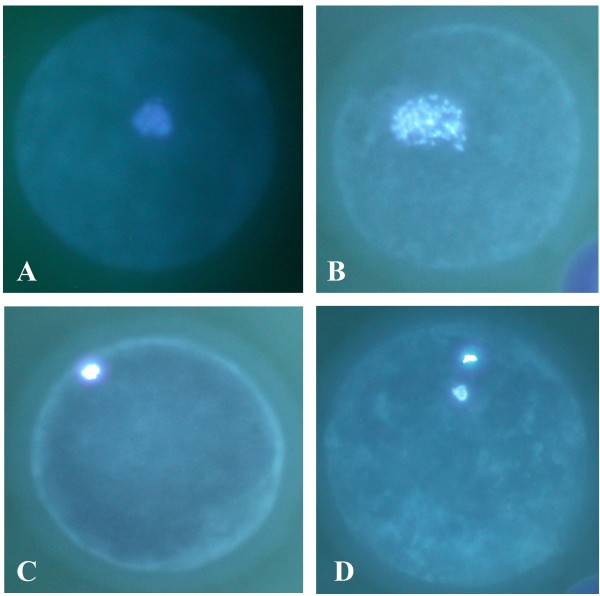
**Nuclear status of canine oocytes**. Stained with Hoechst 33342: (A) GV; (B) GVBD; (C) MI; and (D) MII (400×).

After culturing for 48 h, COCs were denuded by exposure to 1% hyaluronidase for 5 min and gentle pipetting to remove cumulus cells. To analyze meiotic maturation, the denuded oocytes were fixed and permeabilized in Dulbecco's phosphate buffer saline (PBS) containing 3.7% (w/v) paraformaldehyde and 0.1% (v/v) Triton X-100 for 20 min at room temperature. They were washed three times in PBS and then transferred to small drops of PBS supplemented with 90% (v/v) glycerol and 1.9 μM Hoechst 33342, placed on glass slides. The oocytes were overlaid with a coverslip supported by four droplets of vaseline/paraffin. They were examined using a fluorescence microscope with a 355-nm wave length excitation filter. The meiotic stage of IVM oocytes was classified as previously described [15]: germinal vesicle (GV), germinal vesicle break down (GVBD), metaphase I (MI) and metaphase II (MII) (Figure 2).

#### Experiment II: Ultrastructural changes of vitrified-warmed canine oocytes

Based on the results of experiment I that there were no significant differences in rates of nuclear maturation among groups the control and vitrified-warmed groups were included in this study. COCs at 0 h of IVM collected from both groups were fixed in 2.5% glutaraldehyde in 0.1 M sodium cacodylate buffer (pH 7.4) for 120 min at 4°C and washed three times in the same buffer for 10 min at 4°C. Samples were post-fixed with 1% osmium tetraoxide in 0.1 M sodium cacodylate buffer for 1 h at 4°C. They were gradually dehydrated for 10 min twice in each concentration in series of ethanol (35, 50, 70, 80, 90, 95 and 100%) and treated twice for 10 min with propylene oxide, infiltrated with 1:1 propylene oxide/resin in embedding capsules (Electron Microscopy Sciences, Washington, PA, USA) overnight, and finally embedded in fresh resin. Thin sections (60-80 nm) were cut with an ultramicrotome and collected on glass slides or 200-mesh thin bar copper grids. Thin sections were stained with saturated uranyl acetate in 80% methanol and lead citrate. These sections were observed and photographed with a transmission electron microscope at 80 KV.

#### Experiment III: Gene expression of vitrified-warmed canine oocytes

Based on the results of experiment I that there were no significant differences in rates of nuclear maturation among groups the control and vitrified-warmed groups were included in this study. They were collected at 0, 24 and 48 h of IVM to investigate gene expression using RT-PCR. All transcripts of COCs were performed by PCR using the Array Pure Nano-scale Purification Kit (Epicentre, WI, USA). The examined genes were apoptosis related BAX and Bcl2, DNA methyltransferase (Dnmt1), heat shock protein 70 (HSP70), superoxide dismutase1 (SOD1) and glyceraldehyde 3-phosphate dehydrogenase (GAPDH). The amplified PCR products were subjected to electrophoresis for visualization on a 1.5% agarose gel follow by UV analysis using the BioDoc-It System (UVP, Upland, CA, USA). The primers used for RT-PCR analysis are shown in Table 1.

**Table 1:** Primers used for RT-PCR analysis

Gene	Primers	Sequence (5'-3')	Tm (°C)
GAPDH	Forward	TGACGACATCAAGAAGGTAGTGA	61.0
	Reverse	TAGCCAAATTCATTGTCATACCAG	59.4
HSP70	Forward	TGCTGAGGATCATCAACGAG	60.4
	Reverse	GCTTGAACTCCTCCACGAAG	62.4
SOD1	Forward	AGTGGGCCTGTTGTGGTATC	62.4
	Reverse	AGTCACATTGCCCAGGTCTC	62.4
BAX	Forward	TTTGCTTCAGGGTTTCATCC	58.4
	Reverse	CTCACGGGGAGAGTCTGTGT	64.5
Bcl-2	Forward	GGATGCCTTTGTGGAACTGT	60.4
	Reverse	GTGGCAGGCCTACTGACTTC	64.5
Dnmt1	Forward	GTGCCTCCAGGACTTCTCAG	64.5
	Reverse	TCGCATGTTTGAGACTTTGC	58.4

### Statistical analysis

All oocytes were randomly distributed within each experimental group and each experiment was repeated at least three times. Data were arc-sine transformed and then subjected to ANOVA; when necessary, differences were located with Duncan's New Multiple Range Test. Differences of P < 0.05 were considered significant.

## Results

### Experiment 1: The effect of vitrification on nuclear maturation of vitrified-warmed canine oocytes

A total of 284 oocytes were vitrified. After warming, 95.7 and 4.3% were classified as normal and abnormal oocytes, respectively. A total of 292, 209, and 200 oocytes of control, exposed and vitrified-warmed groups were cultured and evaluated for nuclear maturation (Table 2). The percentage of vitrified-warmed oocytes arrested at GV stage (46.6%) was significantly (P < 0.05) higher than those of control (6.2%) and exposure (8.7%) groups. The rate of meiotic resumption (GVBD-MII) of vitrified-warmed oocytes (53.4%) was significantly (P < 0.05) lower than those of control (93.8%) and exposure (91.4%) groups. However, there were no statistically significant differences in rates of GV oocytes reached the MII stage.

**Table 2:** Mean (± SD) nuclear status of control, exposed and vitrified-warmed canine oocytes after in vitro culture for 48 h

Treatment	No. oocytes cultured	Nuclear status (%)				
		GV	GVBD	MI	MII	GVBD-MII
Control	292	6.2 ± 7.4^a^	35.4 ± 18.0	50.1 ± 19.1	8.2 ± 3.7	93.8 ± 7.4 ^a^
Exposed	209	8.7 ± 10.1^a^	34.5 ± 14.2	50.1 ± 10.2	6.7 ± 5.0	91.4 ± 10.1 ^a^
Vitrified-warmed	200	46.6 ± 14.8^b^	18.0 ± 10.0	31.5 ± 2.6	3.9 ± 2.3	53.4 ± 14.8 ^b^

Within a column, percentages without a common letter differed (P < 0.05).

### Experiment 2: Ultrastructure of control, exposed and vitrified-warmed canine oocytes

**Figure 3:**
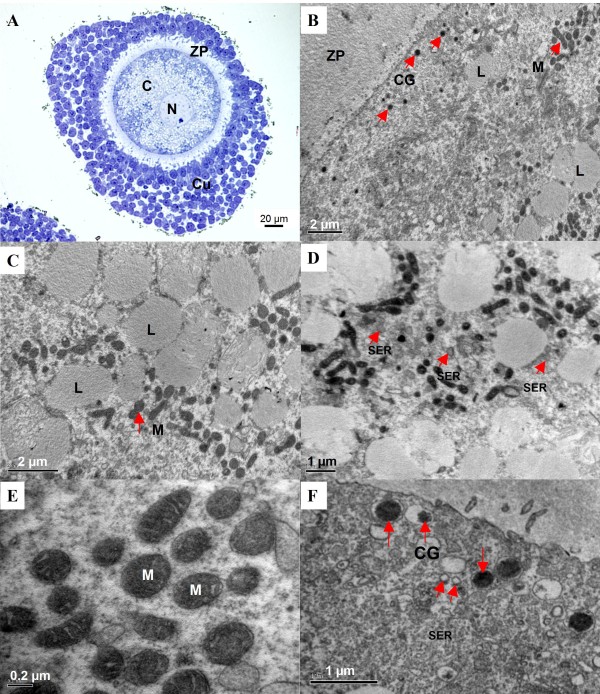
**Transmission electron micrographs of control canine geminal vesicle (GV) oocytes**. A) Semi-thin section of a COC with a central nucleus (N). The cumulus cells densely compact around the oocyte. B) The cortical zone of oocyte, contains a small alignment of cortical granules (CG) beneath the plasma membrane and numerous mitochondria (M). C) The normal shape of lipid droplets (L) and mitochondria (M) scatter in the pericortical cytoplasm of the oocyte. D) Smooth endoplasmic reticulum (SER) are scattered in pericortical cytoplasm. E) Higher magnification of mitochondria showing outer and inner membranes (cristae) with matrix inside. F) Higher magnification of cortical granule (CG) demonstrates the round shape with strong electron dense and smooth endoplasmic reticulum (SER). C = cytoplasm, ZP = zona pellucida, N = nucleus, Cu = cumulus cells, CG = cortical granule, L = lipid droplet, SER = smooth endoplasmic reticulum.

**Figure 4:**
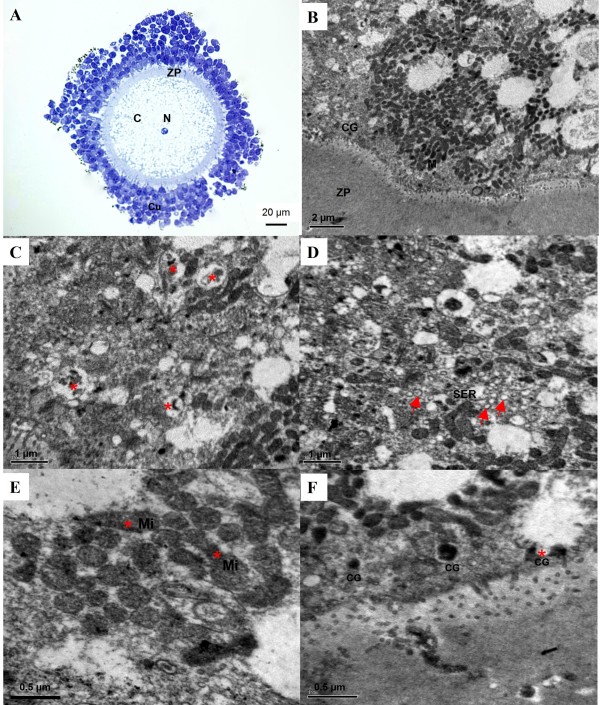
**Transmission electron micrographs of vitrified-warmed canine germinal vesicle (GV) oocytes**. A) Semi-thin section of a COC with a central nucleus (N). The cumulus cells densely compact around the oocyte. B) The cortical zone increases mitochondria (M) numerical density and decrease in cortical granule (CG) numerical density. C) Lipid vesicle turns into smaller droplets with broken membrane and replaced by mitochondria (asterisks). D) Smooth endoplasmic reticulum vesicles (SER) are scattered in pericortical cytoplasm. E) Mitochondria (M) are elongated and of decreased electron density. F) Showing broken CG (asterisk). C = cytoplasm, ZP = zona pellucida, N = nucleus, Cu = cumulus cells, CG = cortical granule, L = lipid droplet, SER = smooth endoplasmic reticulum vesicle.

Transmission electron microscopy (TEM) analysis was performed to investigate the ultrastructural changes of canine oocytes caused by vitrification. A semi-thin section of normal COC showed a large germinal vesicle oocyte with a central nucleus and surrounded with densely compacted cumulus cells (Figure 3A). Figure 3B showed the typical ultrastructure of a normal oocyte at low magnification, which is characterized by numerous mitochondria uniformly scattered within pericortical cytoplasm and a small alignment of cortical granule beneath the plasma membrane. Lipid droplets were of typical round shape (Figure 3C). The smooth endoplasmic reticulum (SER) was concentrated in the perinuclear region and scattered throughout the cytoplasm but in rather low quantities. It was partially or totally surrounded by lipid droplets (Figure 3D). Mitochondria were characterized by a few cristae rarely crossing an electron dense matrix (Figure 3E). They were typically round or elliptical in shape and were distributed around lipid droplets (Figure 3C and 3E). The cortical granules were already present but in highly variable quantities, round in shape and more electron dense. They were either dispersed or grouped in small lines of 3 to 5 granules next to the membrane (Figure 3F). Ultrastructural alterations of oocytes were observed following vitrification. There was an increased mitochondrial density but a decreased cortical granule density at the cortical zone and an irregular plasma membrane (Figure 4B). Lipid droplets became smaller and less electron-lucent. The lipid droplet membranes were broken and mitochondria infiltrated inside lipid droplets (Figure 4C). The smooth endoplasmic reticulum was normal in size but decreased in electron density as compared to the control group (Figure 4D). The mitochondria were elongated and their surfaces became coarse, vague, broken and decreased in electron density (Figure 4E). Furthermore, broken cortical granules were also observed (Figure 4F).

### Experiment 3: Gene expression of control and vitrified-warmed canine oocytes

**Figure 5:**
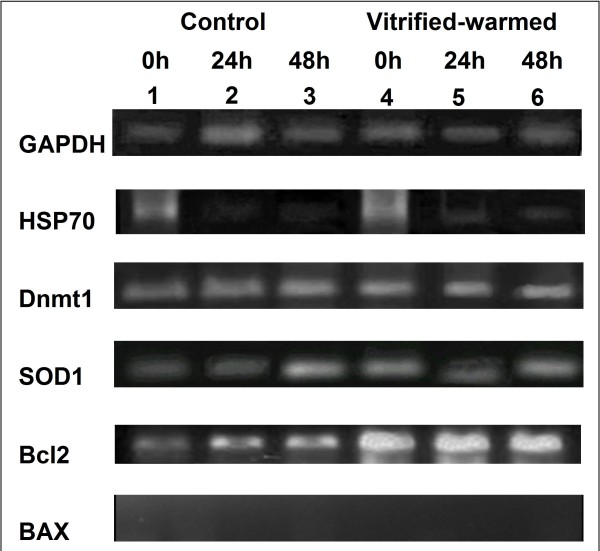
**RT-PCR analysis**. GAPDH, HSP70, SOD1, Dmnt1, Bcl-2 and BAX cDNA expression in control and vitrified-warmed immature oocytes following cultured 0, 24 and 48 h.

In order to assess the effect of vitrification on the expression of stress (HSP70, Dnmt1 and SOD1) and apoptosis-related genes (Bcl2 and BAX), the expression pattern of these selected genes in control and vitrified-warmed oocytes were analyzed (Figure 5). The expression pattern of stress genes (HSP70, Dnnt1 and SOD1) did not differ between control and vitrified-warmed oocytes. HSP70 was strongly expressed at 0 h and decreased over time, whereas Dnmt1 and SOD1 were constantly expressed at 0, 24 and 48 h of cultivation. For apoptosis-related genes (Bcl2 and BAX), Bcl2 was constantly expressed in both groups but strongly expressed in vitrified-warmed group when compared to the control. BAX was absent at 0, 24 and 48 h of cultivation.

## Discussion

Successful cryopreservation of canine oocytes is a stepping stone towards applying assisted reproduction technology (ART) for conservation of endangered canids. To our knowledge, this is the first report conducted on vitrification of immature canine oocytes. Although the rate of meiotic resumption of vitrified-warmed oocytes was significantly lower than those of the control and exposure groups there was no statistically significant difference in rates of meiotic progression to the MII stage.

Various types of cryoprotectants (i.e., EG, DMSO, glycerol, propylene glycol, polyethylene glycol and 1,2-propanediol) have been used in different combinations for the vitrification of mammalian oocytes and embryos [16]. EG has been found to be less toxic than glycerol and propylene glycol to mouse embryos [17]. In addition, it was shown to allow much higher survival rates of bovine embryos [18]. DMSO has been found to be an effective cryoprotectant for vitrification of mouse and hamster oocytes [19,20]. In consequence, EG and DMSO were chosen as cryoprotectants in this present study to avoid ice formation based on the evidence of previous studies in which cryoprotection was useful for the successful vitrification of immature buffalo oocytes [21]. The vitrification technique used high cryoprotectant concentrations, which have been described as toxic to cells [22]. Contrarily, an appropriate phase composition of cryoprotectant mitigates the toxic and osmotic consequences of highly concentrated cryoprotectants [23]. Thus, a mixture of cryoprotectants can decrease individual specific toxicity. In the present study, we vitrified canine oocytes in OPS using mixture of 20% EG and 20% DMSO as previously described [24].

Vitrification of immature bovine and equine oocytes using OPS resulting in subsequent cleavage and development to the blastocyst stage has been reported [25,26]. The OPS method used for cryopreservation of oocytes offers many advantages over other methods. It is simple, inexpensive, achieves a great increase in the speed of cooling by reducing the volume to be vitrified and by thinning the isolating layer between the cooling agent (LN_2_) and the vitrification solution. A further advantage of using very small volumes of vitrified drops was a reduction of the amount of damage to the zona pellucida which occurred during cooling and warming [27].

Our results demonstrated that vitrified-warmed immature canine oocytes in OPS can successfully resume meiosis and develop to the MII stage following IVM. However, the efficiency of oocyte cryopreservation methods is still unsatisfactory. Vitrification procedure reduced oocyte competence to resume meiosis. The assessment of cryo-damaged processes and organelles is fundamental in the evaluation and refinement of current and future cryopreservation protocols. The ultrastructural alterations of vitrified-warmed oocytes revealed by TEM indicated that vitrification procedures affect the pericortical distribution and morphofunctional integrity of cortical granule, mitochondria, lipid droplet and SER.

Morphological examination demonstrated that cortical granules were reduced in numerical density and were damaged in vitrified-warmed oocytes, similar to results in previous studies [12,28,29]. Wessel et al. [30] reported that cortical granules are Golgi-derived, membrane-bound spherical or slightly ovoid organelles formed during the early stages of oocyte and at maturation. Cortical granules are believed to establish the block to polyspermy by preventing penetration of additional spermatozoa. Fuku et al. [28] showed that the numbers of cortical granules along the oolemma were substantially reduced, and fusion of cortical granules with the plasma membrane followed by exocytosis of granule core or intact cortical granule into the perivitelline space was seen in all vitrified bovine IVM oocytes. Ghetler et al. [29] found that the cryopreservation procedure resulted in the loss of cortical granules from the cortical area and in appearance of vesicles within the cytoplasm of both immature and mature human cryopreserved oocytes, which might indicate structural damage occurring from the freezing and warming process.

It has been suggested that the shape and intracellular distribution of mitochondria were related to the level of cell metabolism, proliferation and differentiation, and that these organelles generate the essentials required in a crucial period of the cell cycle [23]. In addition, the present study demonstrated that mitochondria corresponding to the cortical zone were shown to increase in numerical density, to elongate, develop a coarse surface, broken and unclear, of decreased electron density. Similar findings were reported in the exposure of porcine oocytes to vitrification solutions [13]. Roberto et al. [14] reported mitochondria of frozen-thawed human oocytes with decreased matrix electron density or with ruptures of the outer and inner membranes. Mitochondria were the most abundant organelles in mammalian oocytes and their dysfunction or abnormalities would critically determine oocyte and embryo developmental competence. Structural changes to lipid droplets in the present study were in agreement with previous reports in bovine and porcine oocytes [13,31,32]. Xiang et al. [13] suggested that the increased small lipid droplets came from broken larger lipid droplets and exist in the form of smaller drops during vitrification of porcine oocytes. Isachenko et al. also reported that lipid droplets in porcine oocytes changed morphologically during cooling; they changed into a spherical form with lucent streaks [32].

The present study showed the expression of stress genes (HSP70), Dnnt1 and SOD1 were similar in control and vitrified-warmed groups. The identical expression patterns of HSP70, Dnnt1 and SOD1 in the control and vitrified groups, indicating that the vitrification protocol did not alter the expression pattern of these stress genes. It is well known that members of Bcl2 gene family play a major role in regulation of apoptosis. In that regard, Bcl2 is anti-apoptosis and promotes cell survival, whereas BAX is pro-apoptosis and promotes cell death [33]. Apoptosis is an underlying process in oocyte degeneration and embryo fragmentation [34]. This experiment found that Bcl2 was strongly expressed in the vitrified-warmed group when compared to the control, whereas BAX wasn't expressed in both groups. Our results were similar to report in mouse embryos that the expression of BAX gene did not differ from control when morulae were vitrified [35]. Contrarily, Dhali et al. [36] showed that Bcl2 displayed a greater decrease in the vitrified mouse embryos compared to control and expression of BAX gene was down-regulated in the treated group. The expression of Bcl2 was higher in normal than fragmented ones, and the expression did not vary significantly among embryos of varying quality [36]. Moreover, the expression of Bcl-2 was comparatively lower in the vitrified embryos than the normal embryos [34]. Therefore, the differential pattern of gene expression observed in the present study may be used to predict the quality and developmental ability of vitrified immature canine oocytes.

In conclusion, the present study demonstrated that it is possible to cryopreserve immature canine oocyte by vitrification using EG in combination with DMSO and modified OPS protocol. Our results showed that vitrified-warmed immature canine oocytes can resume meiosis and develop to the MII stage. Further studies are required to investigate the fertility and developmental ability of MII oocytes following fertilization.

## Competing interests

The authors declare that they have no competing interests.

## Authors' contributions

BT participated in all aspects of the experiment and writing the manuscript. PS contributed to the RT-PCR analysis. KS and YK designed the experiment and contributed to the analysis and discussion of data. All authors read and approved the final manuscript.
